# Excitation Dependent Phosphorous Property and New Model of the Structured Green Luminescence in ZnO

**DOI:** 10.1038/srep41460

**Published:** 2017-02-02

**Authors:** Honggang Ye, Zhicheng Su, Fei Tang, Mingzheng Wang, Guangde Chen, Jian Wang, Shijie Xu

**Affiliations:** 1 Department of Physics, Shenzhen Institute of Research and Innovation, The University of Hong Kong, Pokfulam Road, Hong Kong, China; 2 Department of Applied Physics, Xi’an Jiaotong University, Xi’an 710049, China

## Abstract

The copper induced green luminescence (GL) with two sets of fine structures in ZnO crystal has been found for several decades (i.e., R. Dingle, Phys. Rev. Lett. **23**, 579 (1969)), but the physical origin of the doublet still remains as an open question up to now. In this paper, we provide new insight into the mechanism of the structured GL band in terms of new experimental findings and theoretical calculations. It is found, for the first time, that the GL signal exhibits persistent afterglow for tens of minutes after the switch-off of below-band-gap excitation light but it cannot occur under above-band-gap excitation. Such a phosphorous property may be interpreted as de-trapping and feeding of electrons from a shallow trapping level via the conduction band to the Cu-related luminescence centers where the Cu^3+^ ion is proposed to work as the final state of the GL emission. From first-principles calculation, such a Cu^3+^ ion in wurtzite ZnO prefers a high spin 3d^8^ state with two non-degenerated half-filled orbitals due to the Jahn-Teller effect, probably leading to the double structures in photoluminescence spectrum. Therefore, this model gives a comprehensively new understanding on the mechanism of the structured GL band in ZnO.

Zinc oxide (ZnO) has re-attracted a great deal of interest due to its outstanding novel properties and promising applications in photonics, spintronics, bio-imaging, photocatalytic and some other fields^1^,^2^,^3^,^4^,^5^,^6^,^7^,^8^,^9^,^10^,^11^,^12^,^13^,^14^,^15^,^16^. Many of these studies have been devoted to the understanding of defects and impurities as well as relevant novel properties of ZnO^3^,^4^,^5^,^7^,^8^,^9^,^10^,^11^,^13^,^14^,^15^. In spite of much effort and great progress, the causes of some basic defects still remain largely a mystery up to date^17^,^18^,^19^,^20^. One of them is the mechanism of the broad green luminescence (GL) band which is commonly seen in the photoluminescence (PL) spectrum of various ZnO including its nanostructures. Native point defects and extrinsic impurities^20^,^21^,^22^,^23^ as well as surface states in nano- and micro-structures^24^,^25^ have been proposed as candidates of GL centers while a unified consensus has not been reached. The sources of GL in ZnO may not be unique, but it was convinced that the trace amount of copper impurity in ZnO (Cu_Zn_) can produce a strong structured GL band^26^,^27^,^28^,^29^,^30^. The copper induced GL has distinguishable fine structures at low temperatures, featured by two sets of periodic structures with a constant energy interval of ~30 meV. It is hence generally referred to as the *structured* GL to distinguish it from the GL bands induced by other defects. Each set of periodic fine structures has been firmly identified as the longitudinal optical (LO) phonon sidebands of Cu related optical transition^31^,^32^. The first zero-phonon line (ZPL) located at 433.5 nm (~2.860 eV) and the spectral center of the band is at ~510 nm (2.431 eV). The overall lineshape of the structured GL band can be well re-produced by using the Multimode Brownian Oscillator (MBO) model with Huang-Rhys factor of 6.5 and phonon energy of ~71 meV (i.e., the characteristic energy of LO phonon in ZnO)^31^,^32^. Nonetheless, the involved electronic levels are not yet settle down, especially the origin of the doublets still remains elusive.

In the earlier article of Dingle^26^, the ground state of the GL band was assigned to the Cu^2+^ ion with 3d^9^ electron configuration, and the excited state was specified to the Cu^1+^ ion with completely filled 3d shell. The luminescence process was described as an internal radiative recombination of the Cu^2+^ ion within hexagonal host lattice^33^. In such a model, the doublet structure was straightforwardly attributed to the copper isotope. However, this point of view can be easily challenged since the energy separation (0.1 meV) of the ZPL lines of ^63^Cu and ^65^Cu was found to be much smaller than the observed one (~30 meV) in the structured GL spectrum^27^,^28^,^29^,^30^,^31^,^32^. An alternative proposal was thus suggested by Reynolds *et al*. that the structured GL was due to a transition from two shallow donors to a deep acceptor^34^. Such an idea was supportable when one considers the behavior of Cu impurity in ZnS, where Cu induced green emission was assigned to the radiative recombination from the conduction band (or shallow donor) to the *t*_2_ level of Cu^2+^ ion^33^,^35^. These un-unified and even controversy arguments motivated further experiments and more rigorous calculations for ascertaining the mechanism and origin of the structured GL emission in ZnO. Such a work is also undoubtedly of broad interest because of its many-body interaction nature and outstanding exampling property.

In this article, we report new important findings and understanding on the structured GL in ZnO. The structured GL was found to show phosphorous property when near-resonance excitation well below the fundamental band gap of ZnO was used. In addition, variable-temperature photoluminescence excitation (PLE) spectra provided more information of the GL band. On the basis of the new experimental results and the first-principles calculations, we propose the high spin 3d^8^ electron states of Cu^3+^ ion having two non-degenerated half-filled orbitals to be responsible for the doublet structure in the GL band. In the new model, the emissive transitions take place from a shallow donor to the split 3d^8^ electron states of Cu^3+^ ion.

The ZnO sample studied in the present work is a bulk crystal rod with hexagonal shape, as shown by the inset in Fig. 1. The sample was grown with hydrothermal method. It is highly transparent, with diameter of ~3 mm and length of ~30 mm. When excited by a 325 nm He-Cd laser at low temperatures the sample shows very strong and sharp band-edge excitonic emission peaks and clear phonon sidebands, as revealed in Fig. 1, indicating its high optical and crystalline quality^31^. Very recently, we have shown that the structured GL band was indeed by Cu impurity in ZnO, either intentionally implanted or traced^30^. The PL and PLE spectra of the sample were carried out on a home-made system^36^.

Detected at 510 nm, the PLE spectra of the sample were recorded at different temperatures ranging from 10 to 310 K, and depicted as a three-dimensional chart in Fig. 2. As seen in Fig. 2(a), besides the band edge peak (the shoulder peaking at 355 nm) the main peak of the PLE spectra centers at 383 nm at low temperatures and red-shifts to 396 nm at high temperatures, well below the fundamental band gap of bulk ZnO^37^. Under the excitation wavelengths of 383 and 396 nm respectively, the photoluminescence (PL) spectra obtained at different temperatures are almost completely the same as those excited by a 325 nm He-Cd laser. The one excited by the 383 nm light at 10 K was illustrated by the green curve in Fig. 1(b). These results indicate that the structured GL can be efficiently excited by the light with photon energies below the band gap (BBG excitation). The BBG excitation of GL in ZnO had been reported in Zn-rich ZnO samples, and manifested as a narrow peak overlapping with the absorption edge^38^,^39^. It was attributed by these researchers to the thermal activation of shallow donors since it was very weak at low temperatures and increased with increasing temperature up to ~180 K. Obviously, our experimental results has distinct origin because the highest BBG PLE peak appears at the lowest temperature used in our study (i.e., 10 K), and it is well separated from the band-edge shoulder. The BBG PLE peak is here attributed to a resonant absorption of the electronic levels involved in the structured GL emission in ZnO. In the MBO model electron-phonon coupling is responsible for the periodic phonon sidebands and broadening. This model also predicts that the phonon-assisted absorption spectrum shall be a mirror image of the PL spectrum relative to the ZPL, as seen by the dashed curve in Fig. 2(b)^31^. Note that PLE spectrum of a real sample is often not equivalent to its absorption spectrum because more physical processes are usually involved within PLE spectrum^40^. In terms of spectral structures, for example, PLE spectrum may lose some absorption structures due to inefficient downward relaxation or efficient transfer of the carriers to other levels. In the case of study here, the PLE spectrum was narrower, obviously missing some higher energy absorption structures. Electrons excited to the higher energy states of the GL centers may efficiently escape to other states such as the host conduction band, causing a narrowed PLE spectrum. The escaped electrons may be described by


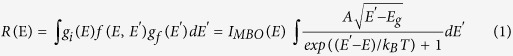


where *g*_*i*_(*E*) is the energy distribution of excited electrons in the luminescence centers, adopting the absorption curve *I*_*MBO*_(*E*) given by the MBO model; *g*_f_(*E*) is the density of states of the conduction band, generally described by 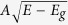, *A* represents a constant, and *f(E, E*′) is in the form of the Fermi-Dirac distribution function. After removing *R(E*) from *I*_*MBO*_(*E*), a modified theoretical absorption curve was obtained and depicted by the solid blue line in Fig. 2(b), which is in better agreement with the measured PLE spectrum. In fact, similar escaping process of excited carriers has been argued in the localized-state-ensemble luminescence model^41^,^42^. Moreover, the escaping probability is usually a strong function of temperature^42^. In this picture, the noticeable redshift of the PLE peak from 383 to 396 nm with temperature could also be well understood.

In addition to the PLE spectral structures, the most amazing finding could be remarkable phosphorous behavior of the structured GL band which exhibits persistent afterglow for tens of minutes after turning off the BBG excitation light, as shown in Fig. 3. However, *this phenomenon did not occur when the photon energy of excitation light was larger than the fundamental band gap of ZnO.* The recorded decaying traces of the PL signal at 510 nm were depicted in Fig. 3 for the excitation wavelength of 383 nm. For clarity, the measured decaying curves were drawn as two groups for T ≤ 75 K in Fig. 3(a) and for T ≥ 75 K in Fig. 3(b). The results were nearly completely repeated for other BBG excitation wavelengths. In this figure the zero time was set at the time when the excitation light was just switched off. The curves of different temperatures were manually aligned by their steady-state intensities. Shown in the inset in Fig. 3(a) is an emission spectrum after time decay of 20 s at T = 60 K. It can be seen that the spectrum of the afterglow keeps its spectral lineshape, i.e., the same as the steady-state GL band, indicating that the afterglow indeed stems from the same luminescence centers except its intensity attenuating.

Each curve in Fig. 3 may be regarded as two time processes, namely a fast decay immediately after the shutdown of excitation followed by a slow process. For T ≤ 50 K, it seems that only the fast decay process was detected. At medium high temperatures, the two time processes can be simultaneously observed. However, when the temperature is increased beyond 120 K, the phosphorescence tends to disappear. The phosphorescence is usually explained in terms of trapping and de-trapping of carriers^43^. Because the intentionally undoped ZnO always shows n-type conductivity, it is thus most likely that trapping and de-trapping of electrons via the conduction band is responsible for the phosphorescence of the structured GL band. When the fast process may be a convolution of intrinsic very fast radiative recombination of carriers with the response time profile of the entire instrument system, the slow decaying curves may accurately reflect a real physical process such as de-trapping or feeding of electrons from a neighboring trapping level or center. It is easy to prove that afterglow from a simple de-trapping process follows a single exponential decay and the decaying time constant *τ* is principally related with the trapping depth Δ*E*, 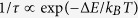, in which *k*_*B*_ is the Boltzmann constant. The trapping depth thus can be derived by fitting the decaying curves at different temperatures. As shown in the inset in Fig. 3(b), the fitting is very well and the obtained trapping depth is 105 meV. For the natural n-type conductivity of ZnO, variety of shallow donors was proposed to be possible origins^3^,^4^,^5^,^18^. Therefore, the existence of electron trapping centers must be a certain conclusion in ZnO. It is interestingly noted that the temperature dependence tendency in the inset in Fig. 3(b) begins to quickly increase for temperature higher than ~90 K. Coincidently, the measured PLE intensity increases again at this temperature, as seen in Fig. 2(a). Such a coincidence strongly suggests that a same physical process happens at ~90 K, which may be the thermal activation of the trapped electrons from the trapping centers and feeding to the luminescent center.

On the basis of above results and arguments, a precise picture showing the distinct mechanism of the structured GL emission in ZnO may be schematically drawn in Fig. 4, where the LO phonon-assisted absorption (upward arrows) and emission (downward arrows) were presented in configuration coordinate diagrams. In this model picture the initial states of the GL emission located near the conduction band minimum (CBM) of ZnO. The main reasons for such justification lie on the fact of the efficient phosphorescence at very low temperatures (e.g. 10 K). Even though the exact energetic location of the pure electronic level cannot be determined, some higher-order vibronic levels (electron-phonon coupling states) shall be significantly overlapping with the conduction band so that efficient electron exchange can take place between the conduction band and the vibronic states. Consequently, the final states involved in the GL emission shall be vibronic levels associated with the 3d orbital of copper impurity. This argument is consistent with the recent density functional theory (DFT) calculations convincingly showing that the 3d orbitals of copper located near the valence band maximum (VBM) of ZnO^44^,^45^,^46^. Although the shallow donor participated in the GL band has not yet been identified, such self-activated configurational coordinate transition has been well documented in ZnS crystal which is another similar II-VI compound^47^, and recently argued to be demonstrated in the Zn-ion implanted ZnO crystal^48^.

The mechanism of above-band-gap excitation is also shown in Fig. 4. The major difference between below- and above-band-gap excitation may fall in this regard that the former generates holes localized nearby Cu impurities while the latter produces movable holes in the host valance band. To produce the characteristic GL in the latter case, the holes generated in the host valance band may have to be captured firstly by the copper impurities. Under this condition, the excited electrons in conduction band may also be trapped by shallow donors, but the holes in valance band can move toward them and recombine with them. That is why the phosphorous phenomenon cannot be seen under above-band-gap excitation. Further supporting evidence for this argument was shown in Fig. 5, where the decaying curves were recorded for phosphorescence process manually disturbed by the 325 nm He-Cd laser pulses for three seconds. It can be clearly seen that the exposure to the 325 nm laser pulse, meaning the temporary above-band-gap excitation, can lead to a pulse-like increase and an abrupt drop in luminescence intensity. After some time the decaying tendency returns to the previous rates. The abrupt drop confirms that the trapped electrons can be eliminated by movable holes in the valance band. The recovery time duration depends on temperature, (i.e., the shaded regions in Fig. 5) becoming shorter for higher temperatures, indicating that the feeding rate of electrons becomes larger with increasing the temperature.

From the mechanism schematically shown in Fig. 4, one can justify that under the BBG excitation or capturing a hole from the valance band the Zn-substituting Cu impurity will change its charge state from Cu^2+^ to Cu^3+^, and possesses a 3d^8^ electronic configuration. DFT based calculation result shows that a high spin state with magnetic moment of 2 *μ*_*B*_ (*μ*_*B*_ is the Bohr magneton) is energy favorable for Cu^3+^ ion in ZnO. It means that there are two unpaired electrons with parallel spin polarizations in the 3d orbitals. The spin polarized energy band structure is illustrated in Fig. 6, in which the orbitals of interest (marked by the green and blue lines respectively) will work as the two empty states. It is noteworthy that the two states are separated. This means that there could be two separate final electronic levels for the GL process, as schematically shown in Fig. 4. They shall be responsible for the doublet structures in the GL band. In fact, the splitting of the two originally degenerate states can be understood in terms of the Jahn-Teller effect. The spatial distributions of the two states are illustrated in Fig. 6(c). Both of them are localized nearby the Cu luminescent center, but exhibit different orientations. Manifested as the Jahn-Teller effect, a geometric distortion of the tetrahedron structure may remove the degeneracy of the two states and induce an energy separation between them. The calculated energy separation is 18 meV, which is smaller than the experimental value (27 meV) derived from the PL spectrum. Considering the complicated many-body nature of the problem and the well-known underestimation of semiconductor band gap with DFT calculation, the agreement between experimental value and DFT calculation result shall be quantitatively good. Here the calculated band gap of ZnO is 2.14 eV which is substantially lower than the fundamental band gap of ZnO^37^. Very recently, a dummy model including a Jahn-Teller effect has been developed by Liao *et al*. for Cu^2+^ ions in biological systems^49^, further showing both ubiquitous and crucial nature of copper ion-related Jahn-Teller distortion in materials science and biology.

This paper reports a new insight into the mechanism of the structured GL band in ZnO. It was found that the copper related structured GL in ZnO can be efficiently excited by the light with photon energy below the fundamental absorption edge of ZnO, and it exhibits an amazing persistent afterglow after switching off such excitation. However, the above-band-gap excitation cannot produce phosphorescence phenomenon, and can critically quench the afterglow. Based on these interesting phenomena and combined with the DFT calculations, the structured GL band in ZnO was attributed to the optical transition from a level involving someone shallow donor near the CBM to the 3d orbitals of Cu impurity at a Zn site. Such a high spin 3d^8^ configuration of Cu impurity may have two non-degenerated half-filled sublevels, being responsible for the doublet structure in the PL spectrum.

## Experimental Section and Methods

The PL and PLE spectra of the sample were carried out on a home-made system composed of two Acton monochromators with focus length of 30 cm^36^. A standard lock-in amplification technique was employed. The light source for PLE measurement was a high-pressure Xe-lamp (Müller, Germany), and the excitation sources for PL spectrum include a 325 nm He-Cd laser and ultra-violet light emitting diodes with emission wavelengths centering at 385 and 395 nm respectively. In the variable-temperature PLE spectral measurements, the rod of ZnO was mounted with silver paint on the cold finger of a Janis closed cycled cryostat.

The DFT-based calculations were performed within the generalized gradient approximation plus U (GGA + U) framework as implemented by the Vienna Ab initio Simulation Package code^50^. The interaction between core and valence electrons is described with the projector augmented-wave potential^51^. We adopt U = 7.5 eV for the semi-core Zn-3d states. A 96 atoms cubic supercell with one Zn atom being replaced by Cu was used to simulate an isolating impurity. The electronic wave function was expanded up to a cutoff energy of 400 eV and we employ Monkhorst-Pack sampling scheme with k-point mesh of 3 × 3 × 3. The atomic positions are allowed to fully relax by minimizing the quantum mechanical force on each ion to be less than 0.03 eV/Å.

## Additional Information

**How to cite this article**: Ye, H. *et al*. Excitation Dependent Phosphorous Property and New Model of the Structured Green Luminescence in ZnO. *Sci. Rep.* **7**, 41460; doi: 10.1038/srep41460 (2017).

**Publisher's note:** Springer Nature remains neutral with regard to jurisdictional claims in published maps and institutional affiliations.

## Figures and Tables

**Figure 1:**
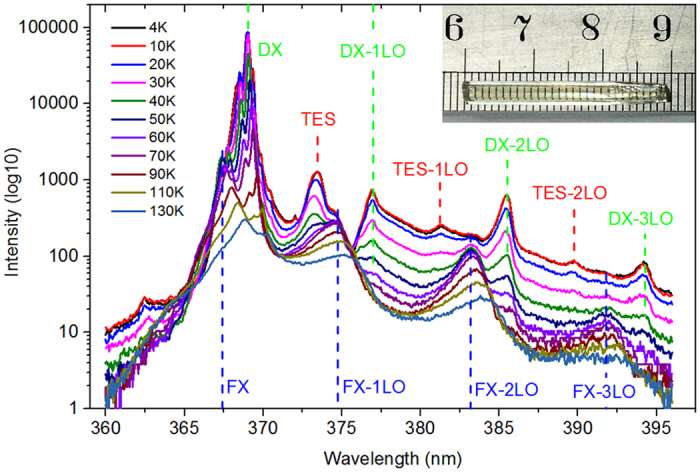
The near band-edge emission peaks from free exciton (FX), donor bound exciton (DX), two electron satellite (TES) recombination and their longitudinal optical (LO) phonon sidebands. The inset is a photo of the studied ZnO rod sample.

**Figure 2:**
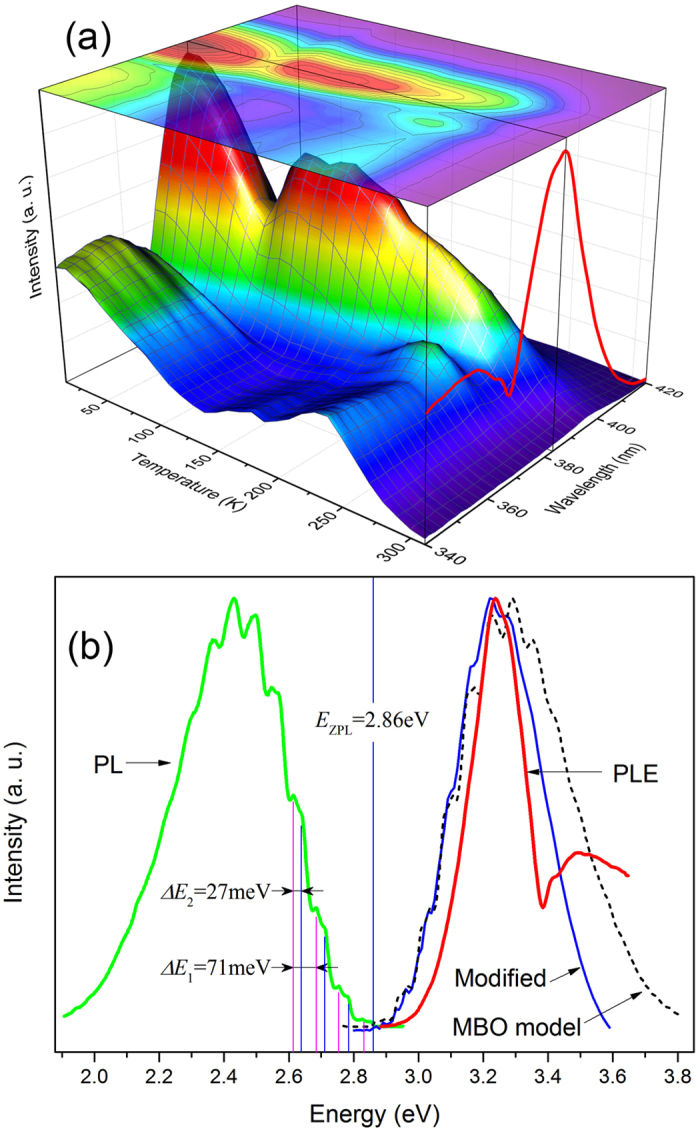
(**a**) Three-dimensional illustration and a plane projection (top contour) of the PLE signal of the sample at different temperatures for detection wavelength of 510 nm. The red curve in the right-hand side plane was the PLE spectrum recorded at 10 K. (**b**) 10 K structured GL (green) and the corresponding PLE spectrum (red). A theoretical absorption spectrum (dashed curve) based on MBO model and a modified one by considering the escaping of excited electrons (blue curve) were also drawn.

**Figure 3:**
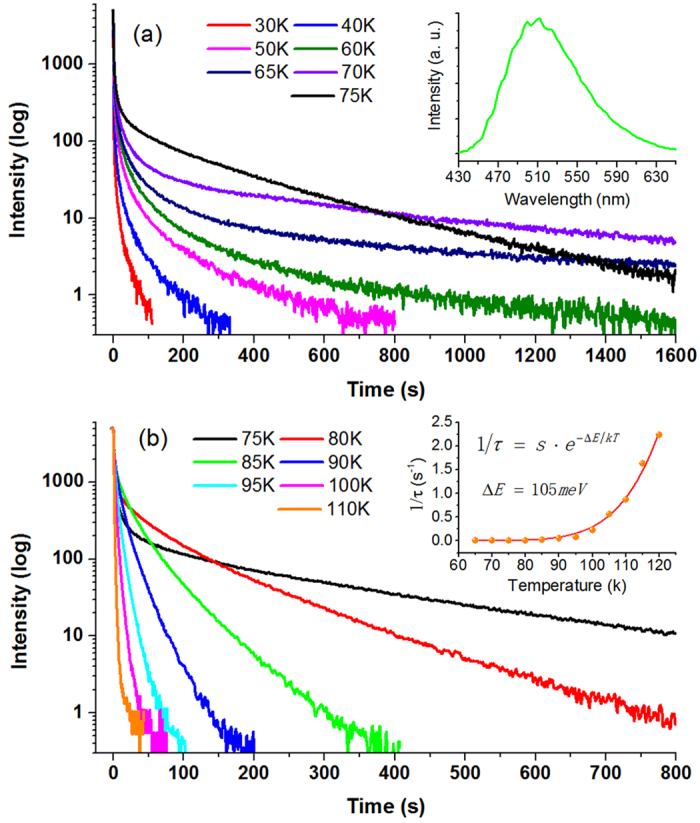
The decaying curves (semi-logarithmical scale) of the ZnO GL recorded at different temperatures for the BBG excitation. The inset in (**a**) shows the PL spectrum registered at 60 K after 20 s time delay, while the inset in (**b**) illustrates the extracted slow time constants (filled circles) vs. temperature. The solid line in the inset represents a fitting curve with a single exponential function.

**Figure 4:**
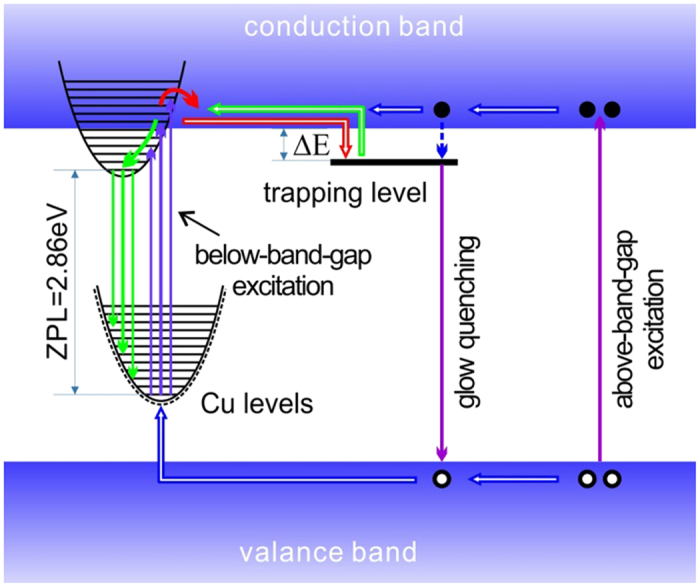
A schematic diagram of the relevant optical transitions and carrier transfer mechanisms of the structured GL band in ZnO under below- and above-band-gap excitation. The phonon assistant absorption (upward arrows) and emission (downward arrows) are shown by configuration coordinate diagrams.

**Figure 5:**
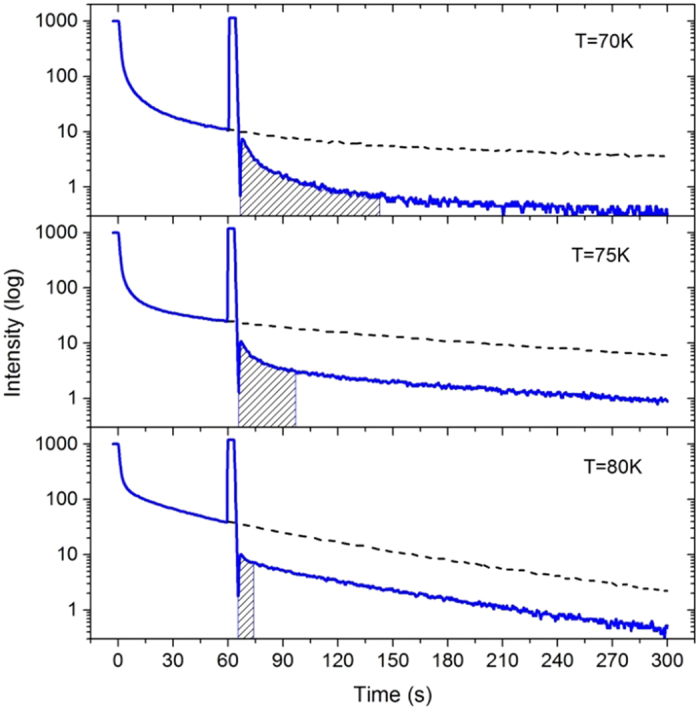
Decaying traces of the GL band at 510 nm disturbed by 325 nm excitation for 3 s were recorded in ZnO at temperatures of 70, 75 and 80 K.

**Figure 6:**
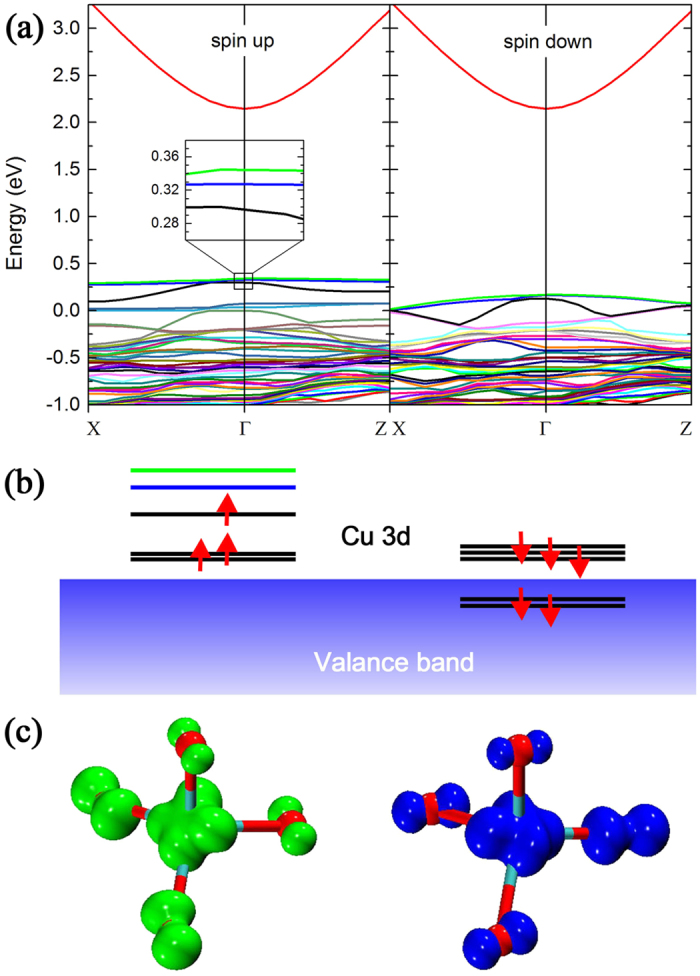
(**a**) The spin polarized energy band structures of Cu^3+^ ion in ZnO. The inset in the spin up band structure shows close up of the Cu 3d-*t*_2_ branch near Γ point. For clarity the relative position of Cu 3d orbitals are drawn schematically in (**b**). (**c**) The spatial distributions of the two empty states marked in green and blue color in (**b**). The red balls represent the oxygen atoms, and the copper atom at the center of tetrahedron is embedded in the isosurface.
